# Scalable Fabrication of Light-Responsive Superhydrophobic Composite Phase Change Materials via Bionic-Engineered Wood for Solar–Thermal Energy Management

**DOI:** 10.3390/molecules30010168

**Published:** 2025-01-04

**Authors:** Yang Meng, Jiangyu Zhang, Yuchan Li, Hui Jiang, Delong Xie

**Affiliations:** Yunnan Provincial Key Laboratory of Energy Saving in Phosphorus Chemical Engineering and New Phosphorus Materials, Yunnan International Joint Laboratory of Sustainable Polymers, The Higher Educational Key Laboratory for Phosphorus Chemical Engineering of Yunnan Province, Faculty of Chemical Engineering, Kunming University of Science and Technology, Kunming 650500, China; zhangjiangyu@stu.edu.cn (J.Z.); liyuchan@stu.edu.cn (Y.L.); jianghui@stu.kust.edu.cn (H.J.);

**Keywords:** wood, phase change materials, superhydrophobicity, solar–thermal conversion

## Abstract

The growing demand for sustainable energy storage solutions has underscored the importance of phase change materials (PCMs) for thermal energy management. However, traditional PCMs are always inherently constrained by issues such as leakage, poor thermal conductivity, and lack of solar energy conversion capacity. Herein, a multifunctional composite phase change material (CPCM) is developed using a balsa-derived morphology genetic scaffold, engineered via bionic catechol surface chemistry. The scaffold undergoes selective delignification, followed by a simple, room-temperature polydopamine (PDA) modification to deposit Ag nanoparticles (Ag NPs) and graft octadecyl chains, resulting in a superhydrophobic hierarchical structure. This superhydrophobicity plays a critical role in preventing PCM leakage and enhancing environmental adaptability, ensuring long-term stability under diverse conditions. Encapsulating stearic acid (SA) as the PCM, the CPCM exhibits exceptional stability, achieving a high latent heat of 175.5 J g^−1^ and an energy storage efficiency of 87.7%. In addition, the thermal conductivity of the CPCM is significantly enhanced along the longitudinal direction, a 2.1-fold increase compared to pure SA, due to the integration of Ag NPs and the unidirectional wood architecture. This synergy also drives efficient photothermal conversion via π-π stacking interactions of PDA and the surface plasmon effects of Ag NPs, enabling rapid solar-to-thermal energy conversion. Moreover, the CPCM demonstrates remarkable water resistance, self-cleaning ability, and long-term thermal reliability, retaining its functionality through 100 heating–cooling cycles. This multifunctional balsa-based CPCM represents a breakthrough in integrating phase-change behavior with advanced environmental adaptability, offering promising applications in solar–thermal energy systems.

## 1. Introduction

Thermal energy remains one of the most extensively utilized energy sources worldwide, accounting for over 50% of total energy consumption [1]. However, the predominant reliance on nonrenewable fossil fuels for thermal energy supply has led to critical challenges, including resource depletion and severe environmental pollution [2]. To address these pressing issues and promote long-term ecological and societal sustainability, transitioning from nonrenewable to renewable and clean energy sources has become imperative [3]. Among renewable options, solar energy is a particularly promising renewable option due to its abundance, wide availability, and environmental friendliness [4]. Nevertheless, the inherent intermittency and variability of solar energy remain significant barriers to its widespread utilization [5,6]. In recent years, the development of advanced energy storage systems has emerged as a crucial strategy for effectively converting and managing solar energy. These systems utilize sensible heat, latent heat, or chemical heat storage to address the imbalance between energy supply and demand [7]. Among these strategies, phase change materials (PCMs) have gained substantial attention due to their ability to store, transfer, and release large amounts of latent heat through phase transitions [8,9]. PCMs offer distinct advantages, including isothermal heat release, high energy storage density, and excellent chemical stability, making them ideal for solar–thermal energy applications [10,11]. Currently, organic PCMs, such as polyethylene glycol, fatty acids, fatty alcohols, sugar alcohols, and their advanced blends or chemically modified derivatives, dominate solar–thermal storage systems, featuring their low supercooling, high latent heat, excellent thermal stability, and non-toxic properties [12]. However, the practical application of organic PCMs in solar–thermal systems faces several challenges, including leakage risks, limited thermal storage efficiency due to their inherently low thermal conductivity, and a lack of integrated solar–thermal conversion capabilities [13,14]. Overcoming these limitations is essential to maximize the performance of PCM-based solar–thermal systems and unlock their potential in advancing sustainable energy technologies.

To address the inherent instability of organic PCMs, integrating a secondary matrix material through adsorption or encapsulation has emerged as a proven strategy for creating shape-stabilized CPCM systems [15,16]. Porous media such as amorphous carbon [17], expanded graphite [18], MOFs [19], mesoporous silica [20], and mineral clays [21,22] (e.g., vermiculite, montmorillonite, sepiolite, and kaolinite), leverage capillary forces and surface tension within their pores to achieve effective PCM encapsulation. Similarly, polymers such as PVA [23], polyurethane [24], and melamine [25] have been widely used to form microcapsules, fibers, and shell-like structures that enhance PCM stability. Despite their advantages, nano/micro porous media typically exhibit low encapsulation capacity, and challenges in fabricating large-scale materials, while the encapsulation strategy always suffers from the intricate synthesis technology, including emulsion polymerization and electrospinning. Recently, three-dimensional (3D) encapsulation matrices (e.g., carbon foams [26], polyurethane foams [27], and aerogels [28]), have demonstrated promising encapsulation efficiencies exceeding 80%. However, the practical implementation of these systems is hindered by the complicated fabrication processes, the high costs of precursor materials such as MXene and graphene, and the reliance on toxic solvents, which undermine their environmental sustainability. Fortunately, the intricate structures observed in biological systems have inspired innovative approaches to designing encapsulation matrices [29]. Morphology genetic materials, a class of bio-based materials derived from natural structures, provide an elegant “top-down” fabrication strategy that eliminates the need for complex manufacturing processes [30]. Among these, wood stands out as a paradigm of natural engineering due to its highly organized structure, featuring hollow tracheids and specialized membranes (e.g., pits that facilitate efficient water and ion transport). Its hierarchical porosity, spanning from the macroscopic to the nanoscale, makes wood a highly promising basic material for functional improvement [31,32]. Beyond its traditional applications in liquid absorption and filtration, wood has demonstrated significant potential as an effective encapsulation matrix for PCMs. To date, various wood species, including balsa [33,34], basswood [35,36], and poplar [37,38], have been utilized as matrices for developing composite PCMs. However, as a naturally hydrophilic biomass material, wood often exhibits interfacial incompatibility with the typically hydrophobic organic PCMs. Although the morphology genetic porosity of wood enables PCM encapsulation, the resulting CPCMs often suffer from structural instability due to interfacial incompatibility. Additionally, delignification, a commonly employed technique to enlarge wood pores, removes lignin while retaining a cellulosic scaffold, further enhancing the hydrophilicity of wood and exacerbating the instability of the as-prepared CPCMs. Moreover, the hydrophilic nature of wood-based matrices also renders composite PCMs susceptible to contamination by mold, dust, and other pollutants, which are difficult to remove and can degrade the CPCMs operating performance. Enhancing the hydrophobicity of wood-based composites, and ideally achieving superhydrophobicity, represents an effective solution to these challenges [39]. This approach will not only mitigate PCM leakage and structural degradation but also impart resistance to contamination, preserving the functionality and ensuring long-term performance in practical applications.

Superhydrophobic wood-based CPCMs exhibit exceptional stability and unique properties such as self-cleaning, anti-fungal, anti-fouling, and anti-corrosion capabilities, offering significant advantages in resisting external environmental fluctuations. To achieve superhydrophobic modification in wood-based composites, several strategies have been proposed. For instance, spraying a composite coating of perfluorodecyltriethoxysilane (PFDS) and SiO_2_ onto basswood/tetradecanol CPCMs has successfully imparted superhydrophobicity [40]. Similarly, polydimethylsiloxane (PDMS)/expanded graphite coatings have been utilized to enhance water-repellent properties [41]. However, these approaches primarily involve the post-treatment of delignified wood-based CPCMs. While effective at modifying CPCMs surface properties, they fail to address the intrinsic hydrophilicity of the wood matrix, which causes interfacial incompatibility with hydrophobic PCMs and undermines long-term material stability. Beyond hydrophilicity, wood as a biomass-based matrix lacks inherent photothermal conversion and thermal conductivity, further limiting its performance in solar–thermal energy storage systems [42]. Incorporating photothermal agents, such as Fe_3_O_4_ [43] or carbon quantum dots [44], directly into PCMs has shown potential to improve photothermal and heat transfer properties. However, these additives often fail to establish strong adhesion with the wood scaffold, leading to agglomeration and performance degradation over extended use due to gravitational forces. Coincidentally, the hydroxyl-rich surface of delignified wood scaffolds offers a promising solution through advanced surface engineering [45]. These hydroxyl groups can serve as reaction sites for grafting hydrophobic polymers, enhancing interfacial compatibility [46]. Additionally, they can act as anchoring points for integrating two-dimensional photothermal conversion materials such as MXene [47] and graphene [48]. Hence, this dual functionality could simultaneously enable interfacial reinforcement, superhydrophobicity, enhanced thermal conductivity, and photothermal conversion capabilities. Nevertheless, there has been little reported research on surface engineering delignified wood scaffolds to achieve comprehensive functionalization of CPCMs, combining interfacial stabilization, superhydrophobicity, thermal conduction, and photothermal conversion.

In this study, a multifunctional wood morphology genetic CPCM was developed using a balsa-derived scaffold as a robust encapsulation matrix, which was surface-engineered through a simple and efficient bioinspired catechol surface chemistry approach. The preparation process involved the selective delignification of balsa wood to create a porous framework, followed by surface modification under mild room-temperature conditions, including oxidative self-polymerization of PDA as an activation platform for in situ reduction of Ag^+^ to Ag nanoparticles (Ag NPs) and octadecyl group grafting. Stearic acid (SA), serving as the primary PCM, was encapsulated into the modified framework using vacuum impregnation, yielding a multifunctional CPCM with exceptional superhydrophobicity, enhanced interfacial bonding, and efficient photothermal conversion capabilities. The delignified balsa wood scaffold retained a highly directional pore structure with excellent permeability. Following surface modification, the scaffold was enriched with Ag NPs and octadecyl functional groups, forming a micro/nanoscale hierarchical superhydrophobic structure. This modification significantly improved the interfacial compatibility between the hydrophilic wood matrix and the hydrophobic SA, ensuring high encapsulation efficiency. Additionally, the PDA coating contributed to enhanced solar energy conversion by leveraging π-π stacking interactions [49] and the surface plasmon effects of Ag NPs [50], achieving high conversion efficiency across the entire solar spectrum. Ag NPs further facilitated thermal conductivity enhancement, while the directional tracheid structure of the wood framework supported efficient unidirectional photothermal conversion and energy storage. Comprehensive evaluations were conducted to thoroughly characterize the balsa-derived CPCMs, including chemical structure, crystalline state, microstructure, encapsulation efficiency, phase-change performance, solar photothermal conversion capabilities, and superhydrophobic properties. As expected, the bionic catechol surface chemistry approach enabled effective multifunctionalization, transforming the balsa-derived CPCMs into a promising candidate for diverse applications, particularly in sustainable energy storage systems.

## 2. Results and Discussion

### 2.1. Balsa-Derived Morphology Genetic Scaffold and Bionic Superhydrophobic Modification

**Figure 1:**
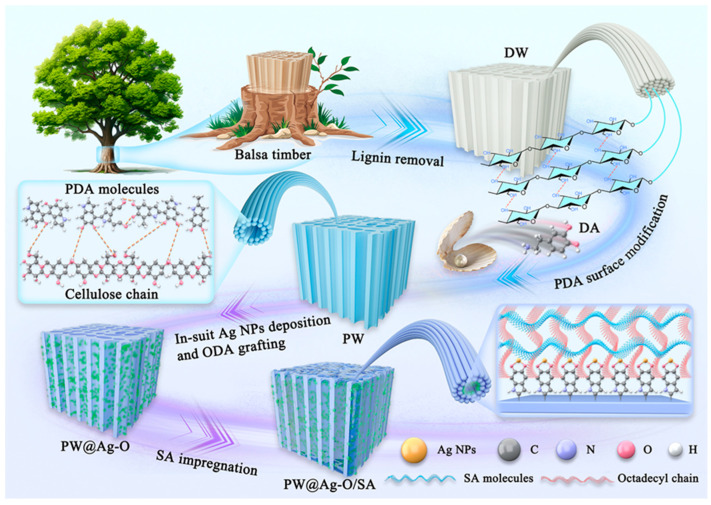
Schematic diagram of the preparation of CPCMs encapsulated by balsa-derived morphology genetic superhydrophobic scaffold.

Figure 1 illustrates the preparation of a superhydrophobic scaffold derived from balsa wood, designed to encapsulate SA for advanced thermal energy storage. Balsa wood, known for its rapid growth and the lowest density among woods (typically below ±87.7 mg/cm^3^), exhibits an ultralight structure with unidirectional tracheids comprising over 90% [51]. This unique cellular architecture has made balsa wood an attractive starting material for sustainable green composite materials. Our prior research demonstrated that selective removal of lignin using an acidic sodium chlorite solution effectively opens clogged pores caused by environmental factors and pests, especially in the latewood regions, which creates a micro-/mesoporous structure with excellent PCM encapsulation capabilities up to 83.5% with PEG [52]. However, the hydroxyl groups exposed on the balsa-derived scaffold merely comprising cellulose and hemicellulose molecules enhance hydrophilicity, which is incompatible with typically hydrophobic PCMs such as paraffin and fatty acid. This incompatibility often leads to leakage of organic PCMs during long-term use, particularly in humid environments. Furthermore, removing the lignin, which contains aromatic structures, results in a white wood-based carrier with reduced visible and UV light absorption, limiting applications in solar–thermal energy conversion [53]. To address these challenges, we adopted a bio-inspired catechol chemistry approach for surface modification [54]. By immersing the balsa-derived scaffold in an alkaline dopamine solution at room temperature, we achieved a catechol-activated surface without the need for stirring or agitation. Especially, the dopamine solution can penetrate the inner pores of the balsa-derived scaffold, spontaneously polymerizing on the internal surfaces under alkaline conditions. The activated balsa scaffold was then used to rapidly reduce Ag^+^ ions, depositing Ag NPs on the surface. Subsequently, ODA was grafted onto the PDA-enriched surface through Michael/Schiff base reactions, forming a multi-layered micro-/nano-structure (Note S2). This simple, rapid modification results in a black PDA-coated wood scaffold, boosting solar absorption through multiple light reflections within its lumens. Studies revealed that the π-π stacking structure of the PDA layer strongly absorbs near-infrared solar wavelengths, while metallic nanoparticles like Ag NPs absorb and convert UV–visible solar energy through surface plasmon resonance effects [55]. Thus, this modification approach achieves full-spectrum solar absorption and conversion. Additionally, the Ag NPs establish conductive pathways within the pore structure, enhancing the thermal conductivity of the balsa-derived scaffold. Finally, grafting ODA and encapsulating SA confer superhydrophobicity to the entire composite, boosting durability and performance in high-humidity or wet conditions.

**Figure 2:**
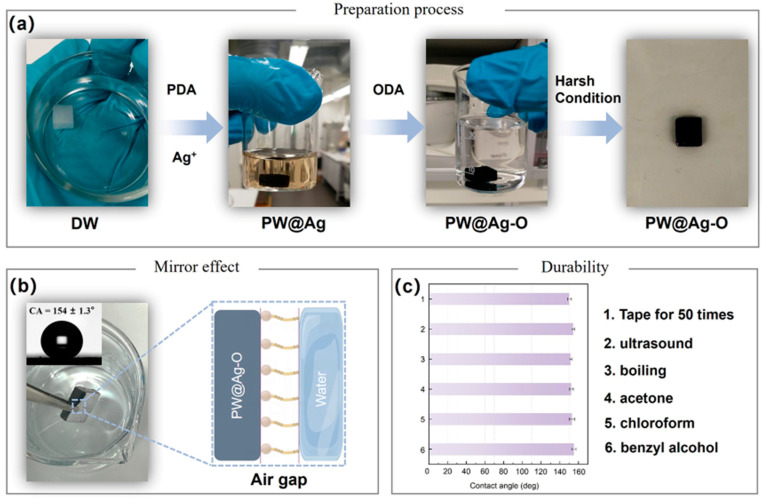
(**a**) Digital photograph of the bioinspired superhydrophobic modification process of the balsa-derived scaffold; (**b**) water-repellent mirror effect on the superhydrophobic balsa-derived scaffold; (**c**) durability of the superhydrophobic interface on the balsa-derived scaffold.

The superhydrophobic modification process of the balsa-derived scaffold is shown in Figure 2a. After lignin removal, DW appears pure white due to the absence of the chromophore groups (e.g., coniferaldehyde). Following catechol-functionalized modification and subsequent reduction to incorporate Ag NPs, DW turns black and becomes fully wetted, sinking to the bottom of an aqueous solution. It is reported that molecules with terminal amino groups can undergo Michael addition and Schiff base reactions with catechol groups under ambient conditions [56]. Accordingly, PW@Ag was immersed in an ethanol solution containing octadecylamine (ODA), enabling the grafting of ODA onto the surface. As shown in Figure 2b, the PW@Ag-O demonstrates pronounced superhydrophobicity, with a contact angle (CA) of 154 ± 1.3° and a distinctive mirror effect when submerged in water. This phenomenon is likely due to the formation of a vacuum-like layer at the surface, created by the combination of micro-/nano-structured Ag NPs and hydrophobic octadecyl groups. This layer effectively prevents direct contact between the water and the balsa-derived scaffold, contributing to its superhydrophobic behavior. Notably, after modification with Ag NPs and superhydrophobic hybridization, the balsa-derived scaffold retains its original black color, which is advantageous for solar–thermal conversion due to its enhanced light absorption. The durability of the superhydrophobic coating under various harsh conditions was also assessed, as shown in Figure 2c. When subjected to a range of harsh conditions such as tap for 50 times, ultrasound, and boiling, as well as immersing in rigorous solvents including acetone, chloroform, and benzyl alcohol, PW@Ag-O maintained a CA consistently above 150°, with minimal variation. Additionally, PW@Ag-O preserved its original black color, indicating that the bionic catechol-based superhydrophobic coating exhibits excellent long-term stability.

### 2.2. Microstructures and Physicochemical Properties of the Balsa-Derived Scaffolds

**Figure 3:**
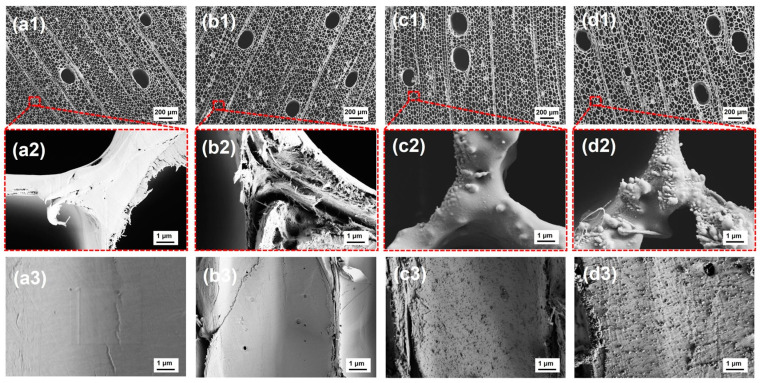
SEM images of balsa-derived scaffolds at different resolutions, showing both cross-sectional views and tracheid surfaces: (**a1**–**a3**) RW, (**b1**–**b3**) DW, (**c1**–**c3**) PW@Ag, and (**d1**–**d3**) PW@Ag-O.

The microscopic morphology of the balsa-derived scaffolds before and after bionic superhydrophobic modification is presented in Figure 3. As illustrated in Figure 3(a1), DW displays the characteristic microstructure of hardwood, featuring aligned lumens formed by abundant axial tracheids with diameters around 30 µm and occasional larger vessels around 200 µm. This pore structure aligns well with the requirements for micro-capillary encapsulation of PCMs, confirming the suitability of the balsa-derived scaffold as an ideal matrix for PCM encapsulation [2]. The removal of lignin hardly disrupts this capillary structure (Figure 3(b1)), as the remaining cellulose and hemicellulose form an interconnected network that supports the rigidity of the balsa cell walls [57]. As expected, after lignin removal, the initially smooth and compact cell walls (Figure 3(a2,a3)) exhibit visible structural changes, with numerous fissures and open pores (Figure 3(b2,b3)). These imperfections enhance the permeability of the balsa wood, facilitating efficient PCM encapsulation, which is consistent with findings in previous studies [51]. Notably, both PW@Ag and PW@Ag-O preserve the structural integrity of balsa wood, including the axial tracheids and vessels (Figure 3(c1,d1)), due to the modifications occurring only on the surface, under room-temperature conditions without harsh organic solvents. Furthermore, the photothermal functionalization through PDA coating and Ag NPs deposition and superhydrophobic functionalization via ODA grafting significantly alter the initially smooth surface. After PDA and Ag NPs modification, the surface roughens, featuring hybrid complexes formed by aggregated PDA and Ag NPs (Figure 3(c2,c3)). These roughened structures, further enhanced by ODA grafting (Figure 3(d2,d3)), create a lotus-leaf-like surface that ensures the superhydrophobicity of the balsa-derived scaffolds, aligning with the phenomena observed in Figure 2b.

**Figure 4:**
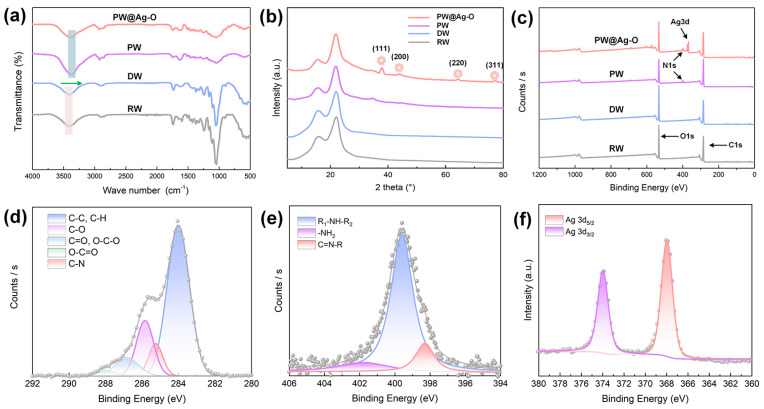
(**a**) FTIR spectra, (**b**) XRD patterns, and (**c**) XPS survey spectra of raw balsa wood (RW), delignified balsa wood (DW), PDA-modified balsa wood (PW), and ODA/PDA/Ag NP hybrid-modified DW (PW@Ag-O). High-resolution XPS spectrum of PW@Ag-O: (**d**) O1s, (**e**) N1s, and (**f**) Ag3d.

The changes in surface functional groups on the modified balsa-derived scaffolds were analyzed through FTIR, as shown in Figure 4a. Balsa wood primarily consists of cellulose, hemicellulose, and lignin. Cellulose exhibits a characteristic peak at 3430 cm^−1^ due to -OH stretching vibrations. Hemicellulose displays strong absorption peaks around 1736 cm^−1^ and 1235 cm^−1^, corresponding to the stretching vibrations of carbonyl (C=O) and acyl groups (CO-OR), respectively [30]. Lignin, on the other hand, has weaker peaks at 1590 cm^−1^, 1505 cm^−1^, and 1462 cm^−1^, associated with aromatic ring vibrations [31]. As seen in Figure 4a, the DW sample reveals only the characteristic peaks of cellulose and hemicellulose, verifying the selective removal of lignin. After catechol-based functionalization, the -OH peak at 3430 cm^−1^ shifts to lower wavenumbers, likely due to hydrogen bonding between catechol groups and cellulose [58]. Additionally, a prominent peak at 1590 cm^−1^ appears, attributed to the aromatic ring vibrations in the PDA molecules. These spectral changes confirm the successful introduction of a PDA coating on the DW surface through a bionic self-polymerization method. After Ag NPs deposition and ODA grafting, the positions and intensities of these functional groups remain unchanged, confirming that the superhydrophobic hybrid modification preserves the primary structure of the PDA coating. Since inorganic peaks are less prominent in FTIR, XRD analysis was conducted on PW@Ag-O. The XRD patterns in Figure 4b show that RW exhibits typical cellulose peaks at 2θ values of 16.0°, 22.0°, and 34.8°, corresponding to the (101), (020), and (040) planes characteristic of type I natural cellulose [33]. These peaks remain visible across all modification stages, indicating that neither lignin removal nor subsequent superhydrophobic treatments compromise the crystalline structure of the cellulosic scaffold, consistent with SEM observations. Notably, the PW@Ag-O sample shows new peaks at 2θ values of 38.1°, 44.2°, 64.4°, and 77.3°, corresponding to the (111), (200), (220), and (311) planes of Ag NPs [50]. This indicates that Ag^+^ ions were successfully reduced to Ag NPs by PDA, and the structure of the Ag NPs remains intact after ODA modification. This stability suggests that the ODA grafting reaction occurs solely on the catechol groups of the PDA without affecting the Ag NPs.

To identify the bonding elements and configurations in the balsa-derived scaffolds before and after modification, XPS analysis was conducted, with peak differentiating and imitating applied to the spectra of specific elements such as C, N, and Ag. In the XPS survey spectra (Figure 4c), only the C1s peak at 285 eV and the O1s peak at 530 eV appear for both RW and DW. After PDA modification, an N1s peak emerges at 402 eV in the PW sample, attributed to the nitrogen-containing PDA, indicating successful catechol functionalization [59]. In the PW@Ag-O sample, an enhanced N1s peak and the appearance of the Ag3d peak at 380 eV confirm the successful spontaneous reduction of Ag^+^ to Ag NPs and the subsequent ODA grafting. As shown in Figure 4d, the C1s spectrum of PW@Ag-O consists of components at 283.5 eV (C-C/C-H), 285.7 eV (C-O), 286.7 eV (C=O/O-C-O), 288.2 eV (O-C=O), and 285.4 eV (C-N), which are characteristic of wood materials [60]. The presence of the C-N structure observed in the XPS analysis indicates the possible contribution from both the PDA molecules and the grafted ODA, supporting the successful modification of the scaffold’s surface. As shown in Figure 4d, the N1s spectrum reveals peaks at 401.6 eV (primary amine, -NH_2_), 399.5 eV (secondary amine, -NH-), and 398.2 eV (C=NR) [61]. The -NH_2_ signal likely results from unreacted ODA, while the -NH- signal may arise from both PDA and the Michael addition reaction with ODA. The C=NR peak indicates a Schiff base reaction between ODA and PDA structures. In addition, as shown in Figure 4f, the Ag3d spectrum of PW@Ag-O shows distinct Ag (3d_5_/_2_) and Ag (3d_3_/_2_) peaks, confirming the presence of metallic silver, which aligns with the XRD results.

Overall, the bionic catechol chemistry method effectively imparts photothermal and superhydrophobic properties to the delignified balsa wood while preserving its aligned pore structure. This will enable effective PCM encapsulation, advancing its use as a high-performance substrate for solar–thermal energy management.

### 2.3. Balsa-Based CPCMs and the Interfacial Enhancement Behavior

**Figure 5:**
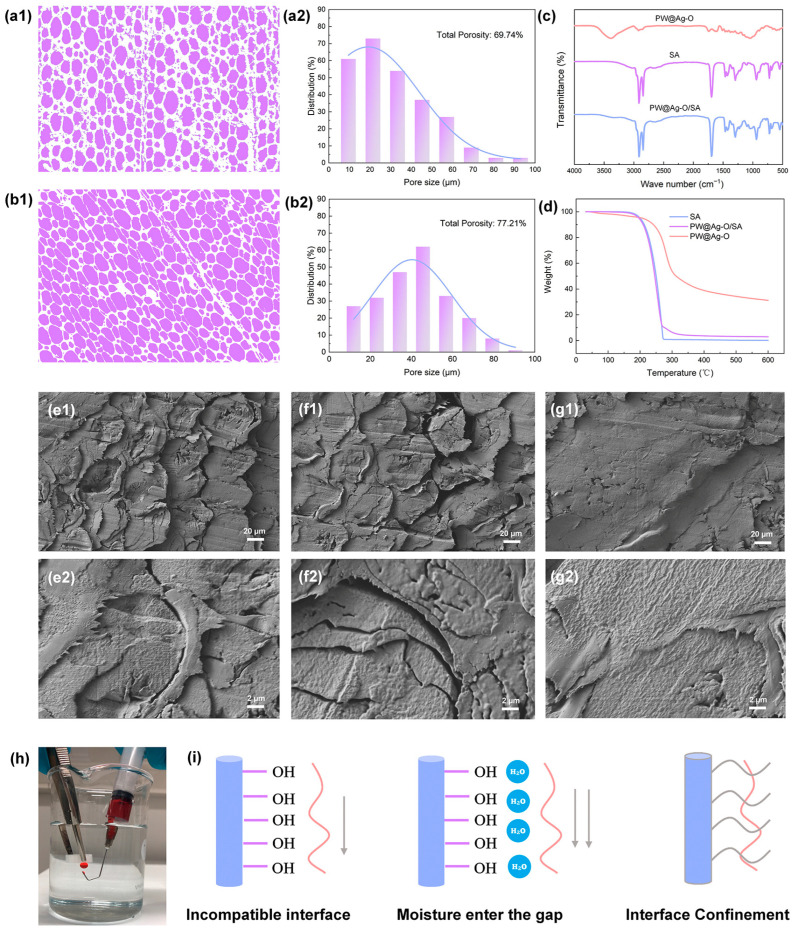
(**a1**) Binary distribution map and (**a2**) pore size distribution statistics of the RW cross-sectional microtopography; (**b1**) binary distribution map and (**b2**) pore size distribution statistics of the PW@Ag-O cross-sectional microtopography; (**c**) FTIR spectra and (**d**) TGA curves of pure SA, modified balsa-derived scaffolds, and their composites; microstructures of CPCMs at different resolutions: (**e1**,**e2**) DW/SA, (**f1**,**f2**) hygroscopic DW/SA, and (**g1**,**g2**) PW@Ag-O/SA; (**h**) digital image of underwater superoleophobic test of DW; (**i**) schematic of interfacial enhancement mechanism in CPCMs.

Figure 5(a1,b1) show the binary separation images of the pore and cell wall structures in the axial tracheids of RW and PW@Ag-O samples. In these images, the light purple areas represent pores, while the white areas represent cell walls. It is evident that the width of the white regions (cell walls) between the purple-marked pores is smaller in PW@Ag-O than in RW, indicating that the delignification process reduces cell wall thickness by removing amorphous components. In addition, the subsequent hybrid modification only occurs at the interface with the micro-nano scale, which will have little effect on cell wall thickness at the macro scale. Digitized binary image analysis provided pore size distribution data, as seen in Figure 5(a2,b2). The pore size distribution in RW ranges from 10 to 80 µm, with a concentration between 10 and 30 µm, reflecting the inherent variability of natural wood [52]. Interestingly, it can be observed the delignification and subsequent hybrid modification narrow the pore size distribution toward ~50 µm, with an increase in porosity from 69.74% (RW) to 77.21% (PW@Ag-O). Studies indicate that a pore size of around 50 µm optimally supports PCM encapsulation, minimizing material loss due to insufficient capillary forces [7]. Thus, delignification and hybrid modification could effectively enhance PCM adsorption efficiency. Using a simple vacuum impregnation method, SA was easily encapsulated within the modified balsa-derived scaffolds, forming stable composite PCMs. The FTIR spectra of the balsa-derived encapsulation scaffolds, SA, and the resulting CPCMs are shown in Figure 5c. All characteristic peaks of SA and PW@Ag-O appear in the composite PW@Ag-O/SA, with no new peaks observed. This indicates that the interaction between SA and the wood scaffold, along with the hybrid surface structure, is purely physical, preserving the mechanical and phase change properties of both the encapsulating scaffold and the PCM. Moreover, thermal stability in operational environments is also critical for evaluating the performance of CPCMs. As shown in Figure 5d, PW@Ag-O exhibits no decomposition below 200 °C, well within the theoretical phase transition temperature of SA [62]. For the composite PCM, rapid thermal decomposition above 150 °C is primarily due to the degradation of encapsulated SA. However, the composite PCM remains stable below 100 °C, retaining its original structure and properties. Therefore, the prepared CPCM demonstrates adequate thermal stability within its intended operational range, meeting the requirements for low-temperature thermal energy storage applications.

Figure 5e–g illustrate the microstructure of DW and PW@Ag-O after encapsulation with SA to form CPCMs. As shown in Figure 5(e1), SA completely fills the micro-capillary lumens of DW after vacuum impregnation, held in place by capillary forces. However, in the high-resolution image (Figure 5(e2)), gaps are observed at the interface between SA and DW cell walls. This may result from the hydrophobic nature of SA and its poor compatibility with the hydrophilic cellulose molecules of DW. Upon moisture exposure and the heating–cooling cycle, it is evident that although SA remains within DW (Figure 5(f1)), the interfacial gaps continue to grow larger, indicating that hydration worsens compatibility. To investigate this phenomenon, the underwater oleophobicity of hydrated DW was tested. As shown in Figure 5h, hydrated DW exhibits strong underwater oleophobicity, repelling stained oil droplets that retain a spherical shape and quickly rise to the surface. This experiment demonstrates that the superoleophobic properties of the hydrated DW could lead to leakage of molten SA. In contrast, the hydrophobic PW@Ag-O scaffold shows strong superhydrophobicity, resisting moisture absorption. The presence of octadecyl groups provides high compatibility with octadecanoic acid, both of which have similar alkyl chains. As observed in Figure 5(g1,g2), SA is securely encapsulated within PW@Ag-O, with no visible distinction between the encapsulant and the scaffold. Based on these microstructural observations, the encapsulation mechanism of SA within the modified balsa-derived scaffold is illustrated in Figure 5i. In DW, the abundant surface hydroxyl groups exhibit poor compatibility with hydrophobic SA, resulting in potential leakage during phase transitions. This incompatibility is further aggravated upon hydration, leading to increased leakage risks in outdoor environments, particularly under humid or rainy conditions. Conversely, the hydrophobic PW@Ag-O scaffold effectively encapsulates SA through a combination of mechanical interlocking and enhanced interfacial interactions, significantly reducing leakage even in the molten state. The superhydrophobicity of PW@Ag-O, coupled with robust interfacial adhesion, provides exceptional environmental resistance, ensuring stable encapsulation of SA and reliable phase change functionality under challenging conditions.

### 2.4. Phase Change Behavior and Thermal Repeatability of Balsa-Based CPCMs

**Figure 6:**
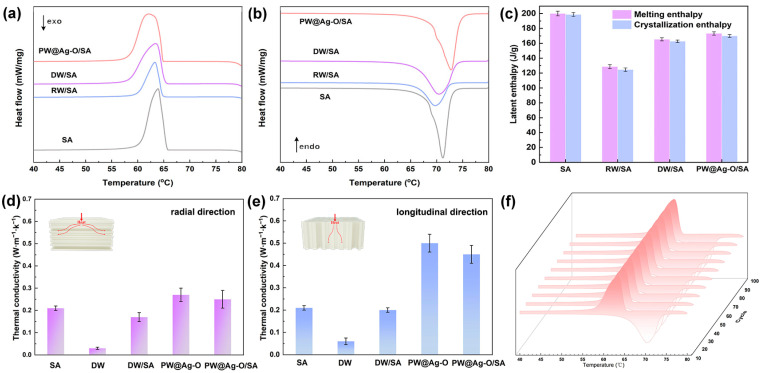
Pure SA and balsa-based CPCMs: (**a**) DSC thermograms during the cooling process; (**b**) DSC thermograms during the heating process; (**c**) statistical analysis of phase-change enthalpy; (**d**) long thermal conductivity; (**e**) longitudinal thermal conductivity; and (**f**) DSC thermograms of PW@Ag-O/SA over 100 thermal cycling tests.

Effective thermal energy management necessitates CPCMs that deliver on three essential fronts: high latent heat storage capacity, an optimal phase transition temperature, and a structurally robust three-dimensional framework. To comprehensively analyze their thermal performance and phase transition behavior, differential scanning calorimetry (DSC) was employed. The DSC curves, presented in Figure 6a,b, illustrate the endothermic and exothermic profiles of pure SA alongside CPCM variants (e.g., RW/SA, DW/SA, and PW@Ag-O/SA). Key thermal parameters, including melting temperature (*T*_m_), melting enthalpy (Δ*H*_m_), freezing temperature (*T*_c_), and crystallization enthalpy (Δ*H*_c_), are systematically summarized in Table S1. Moreover, the energy storage efficiency (*E*) was calculated using Equation (1) to elucidate the effect of the encapsulation framework on phase change performance [40].

(1) $$E=\frac{∆H_{CPCMs}}{∆H_{SA}}\times 100\%$$

where Δ*H_CPCMs_* represents the enthalpy value of the CPCM, and Δ*H_SA_* represents the enthalpy value of pure SA.

As shown in Figure 6a,b, pure SA exhibits sharp melting and crystallization peaks, with a single characteristic peak in both the heating and cooling stages, consistent with the typical DSC curves of SA. As anticipated, after encapsulation within the balsa-derived scaffolds, all CPCMs, including RW/SA, DW/SA, and PW@Ag-O/SA, retain the characteristic DSC features of SA and exhibit similar phase change temperatures. This confirms that the balsa wood scaffolds effectively encapsulate SA while preserving its phase change properties without altering its chemical structure. It is reported that an ideal CPCM should minimize the reduction in phase change enthalpy caused by the encapsulation matrix [15]. As shown in Figure 6c, pure SA exhibits high melting (200.1 J/g) and crystallization (198.8 J/g) enthalpy values, indicative of its excellent thermal energy storage capacity. In the RW/SA system, the obstructed pore structure of RW results in poor encapsulation performance, leading to significantly reduced melting (131.7 J/g) and crystallization (130.8 J/g) enthalpies. After delignification, the enhanced pore openness and permeability of DW substantially improve encapsulation, increasing the melting and crystallization enthalpies to 168.7 J/g and 167.6 J/g, respectively. Notably, the hybrid-modified scaffold, PW@Ag-O/SA, achieves the highest phase-change performance, with melting and crystallization enthalpies of 175.5 J/g and 174.3 J/g, respectively. Moreover, the energy storage efficiency (*E*) was calculated for the composites, yielding values of 65.8%, 84.3%, and 87.7% for RW/SA, DW/SA, and PW@Ag-O/SA, respectively. These results closely align with the actual encapsulation efficiencies obtained through leakage tests, which were 66.3%, 84.7%, and 88.1%, respectively. The observed difference between theoretical and actual enthalpy values is likely attributed to confinement effects at the matrix-PCM interface, where the restricted movement of the PCM reduces the overall enthalpy [63]. However, in the case of wood-based scaffolds, the high SA content minimizes this confinement effect, resulting in a closer match between theoretical and actual values. Interestingly, the enhanced performance of PW@Ag-O/SA is attributed to the hydrophobic octadecyl groups introduced during modification, which form a barrier at the interface between the matrix and SA. This barrier reduces interactions between the carboxyl groups of SA and the hydroxyl groups of DW, thereby weakening hydrogen bonding at the interface. As a result, the octadecyl-modified scaffold enables greater molecular freedom for SA during phase transitions, ensuring that its phase-change properties are preserved. As a result, this interfacial compatibility not only enhances the stability of encapsulated SA but also minimizes leakage, even under operational conditions, making PW@Ag-O/SA highly effective for thermal energy storage applications.

The thermal conductivity of CPCMs is a critical factor in determining their efficiency in heat storage and release, making it essential for these materials to respond rapidly to environmental temperature changes during practical use. As shown in Figure 6d,e, DW exhibits poor thermal conductivity due to the inherently low conductivity of its cellulose framework as well as the highly opened porous structure [53]. The anisotropic nature of balsa wood results in a thermal conductivity of 0.08 W·m^−1^·K^−1^ along the longitudinal direction and 0.06 W·m^−1^·K^−1^ in the radial direction. Pure SA, as an organic polymer, has a relatively low thermal conductivity of 0.22 W·m^−1^·K^−1^. When encapsulated with DW, the composite exhibits a slight reduction in thermal conductivity along the tracheid growth direction, likely due to the inherently low conductivity of the cellulosic scaffold. However, as anticipated, hybrid modification with Ag NPs significantly improves the thermal conductivity of PW@Ag-O. The uniform dispersion of Ag NPs along the cell wall surfaces of the balsa wood framework creates effective pathways for heat transfer, leading to a marked increase in the conductivity of the modified CPCM [55]. Although the encapsulation of PCMs causes a slight reduction in thermal conductivity, the composite materials still maintain relatively high levels. Compared to pure SA, the thermal conductivity of the composite along the longitudinal direction increases by 2.1 times, significantly enhancing the efficiency of heat storage and release. In contrast, the radial thermal conductivity of the composite remains similar to that of pure SA, ensuring that heat is primarily and rapidly transferred along the longitudinal direction while minimizing heat dissipation in the transverse direction, which will enhance the directional heat transfer efficiency. In addition, the thermal reliability of the PW@Ag-O/SA was assessed through 100 heating–cooling thermal cycling tests. As shown in Figure 6f, the DSC curves recorded before and after cycling reveal negligible variations in both phase transition temperature and latent heat of fusion, with only a minor reduction of less than 1 J/g. These results demonstrate the exceptional thermal stability and durability of the balsa-based CPCMs under extended thermal cycling, underscoring their suitability for long-term applications in thermal energy management.

### 2.5. Solar-to-Thermal Energy Conversion and Storage of Balsa-Based CPCMs

**Figure 7:**
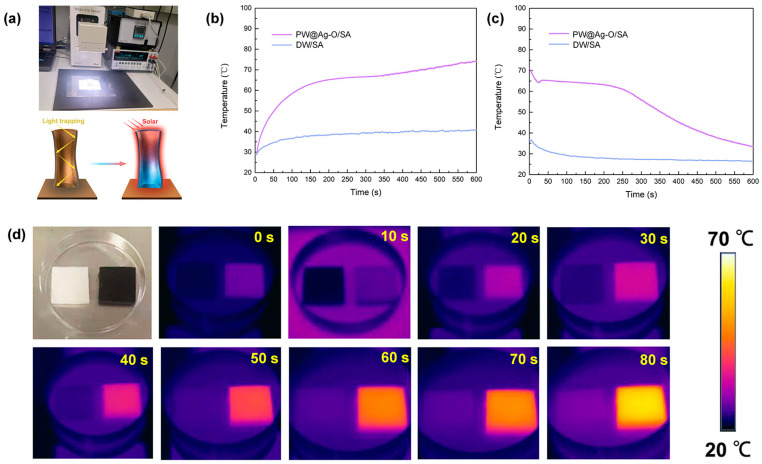
(**a**) Digital image of the photothermal conversion testing system for balsa-based CPCMs and the mechanism of photothermal conversion pathways; (**b**) heating and (**c**) cooling profiles of DW/SA and PW@Ag-O/SA during solar–thermal energy utilization; (**d**) infrared thermographic images of DW/SA and PW@Ag-O/SA under one-sun irradiation.

The photothermal conversion performance of balsa-based CPCMs is crucial for their application in solar–thermal energy systems. A custom-built solar–thermal simulation testing system was developed, in which a 1000 W/m^2^ xenon lamp light source was used to simulate one-sun irradiation (AM 1.5G) by continuously illuminating the CPCMs during testing, as shown in Figure 7a. Temperature changes in DW/SA and PW@Ag-O/SA were monitored using a temperature inspection instrument equipped with PT100 micro-sensors. The recorded data for heating and cooling are presented in Figure 7b,c. Under simulated solar irradiation, the hybrid-modified CPCM, PW@Ag-O/SA, containing Ag NPs and PDA coatings, reached its phase-change temperature of 67.87 °C in approximately 150 s. This temperature remained stable for around 200 s, indicating the conversion of solar energy into latent heat. Subsequently, the temperature began to rise steadily and reached saturation at approximately 74.6 °C. These results align with the infrared thermographic images shown in Figure 7d. The rapid and efficient photothermal conversion performance of PW@Ag-O/SA can be explained by the schematic in Figure 7a. The unidirectional lumen structure of the balsa-derived morphology genetic scaffold facilitates multiple refractions of sunlight, increasing the interaction between solar radiation and the hybridized Ag NPs and PDA coatings on the cell walls. This facilitates efficient photothermal conversion while the Ag NPs create conductive pathways, enabling rapid heat transfer to the encapsulated SA for storage. By contrast, DW/SA exhibited limited photothermal performance, reaching a maximum temperature of 36.3 °C within 100 s of irradiation, after which no further temperature increase was observed. This is attributed to the delignified balsa-derived scaffold mainly containing cellulosic composition, which exhibits high radiative cooling properties but lacks effective photothermal conversion mediators [53]. Following the cessation of xenon lamp irradiation, the cooling performance of the CPCMs was evaluated. For PW@Ag-O/SA, an isothermal heat release phase began at 30 s and lasted approximately 220 s, corresponding to the latent heat released during the phase-change process. In comparison, DW/SA exhibited a rapid temperature drop from 36.3 °C to 27.0 °C within the first 100 s, after which the temperature remained constant. This behavior is due to the absence of a phase-change process in DW/SA, resulting in no latent heat storage or release. Overall, the hybrid modification with Ag NPs and PDA significantly enhances the photothermal conversion efficiency of PW@Ag-O/SA, enabling it to effectively convert solar energy into thermal energy and store it within the PCM. This stored energy can then be rapidly and efficiently released when required, demonstrating the potential of PW@Ag-O/SA for solar–thermal energy applications.

### 2.6. Superhydrophobic Properties and Applications of Balsa-Based CPCMs

**Figure 8:**
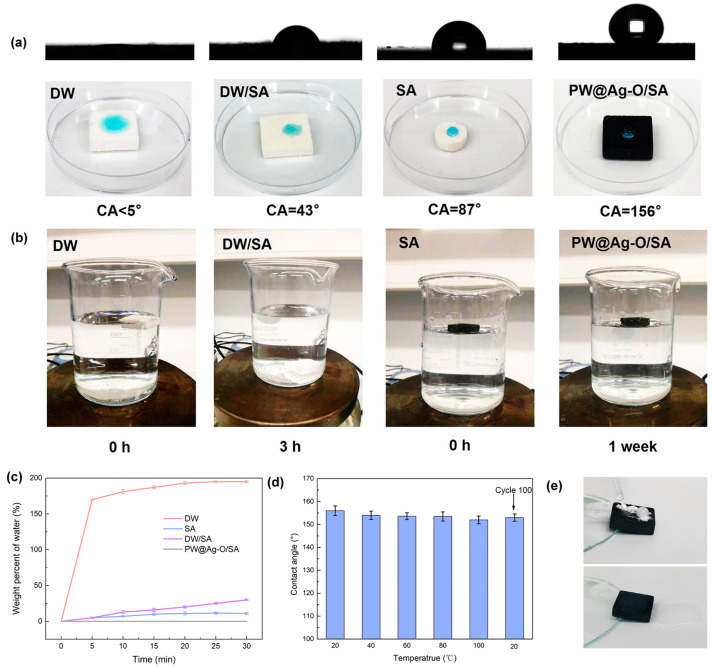
(**a**) Water contact angles and corresponding digital photographs of DW, SA, DW/SA, and PW@Ag-O/SA; (**b**) digital photographs of water absorption experiments for DW/SA and PW@Ag-O/SA; (**c**) water absorption curves of DW, SA, DW/SA, and PW@Ag-O/SA; (**d**) water contact angles of PW@Ag-O/SA under different operating temperatures and after 100 cycles; (**e**) self-cleaning behavior of PW@Ag-O/SA captured in digital images.

The water resistance of balsa-based CPCMs plays an important role in practical applications, ensuring stable PCM encapsulation and long-term durability. It is reported that achieving a superhydrophobic state not only prevents water absorption but also endows the CPCMs with self-cleaning properties, allowing for easy removal of surface contaminants such as dust, thereby maintaining optimal solar–thermal conversion efficiency [64,65]. Water CA measurements for DW, pure SA, DW/SA, and PW@Ag-O/SA were conducted using a video contact angle meter, as shown in Figure 8a. DW exhibited strong hydrophilicity, with water droplets quickly absorbed into the surface, resulting in a CA of less than 5°. The DW/SA composite retained hydrophilic behavior due to interfacial gaps between SA and DW, with droplets slowly absorbed into the encapsulating scaffold, yielding a CA of 43°. Pure SA, with its alkyl groups, showed moderate hydrophobicity, maintaining droplets on the surface with a CA of 87°. In contrast, PW@Ag-O/SA demonstrated superhydrophobic properties, with droplets forming nearly perfect spheres on the surface and a CA exceeding 156°, indicating that the hybrid modification effectively preserved superhydrophobicity after SA encapsulation. Furthermore, the water CA of PW@Ag-O/SA consistently exceeded 150° across a wide operating temperature range of 20 °C to 100 °C, as well as after 100 heating and cooling cycles, demonstrating exceptional environmental stability of its superhydrophobic properties (Figure 8d).

To evaluate water resistance, water absorption experiments were conducted for the balsa-based CPCMs, as shown in Figure 8b. DW/SA, when placed in deionized water, initially floated but became fully submerged within hours, sinking to the bottom of the container after just 3 h. This behavior is likely due to the incompatibility between SA and DW, allowing water to infiltrate interfacial gaps and be absorbed by the hydrophilic DW scaffold, as observed in SEM images (Figure 5(f2)). Conversely, PW@Ag-O/SA remained entirely buoyant at the surface even after one week, demonstrating excellent water resistance. Water absorption capacities for DW, SA, DW/SA, and PW@Ag-O/SA were statistically analyzed, as shown in Figure 8c. Notably, DW exhibited remarkable water absorption, retaining nearly 200 times its weight in water, while pure SA showed moderate water absorption, saturating at approximately 13%. The DW/SA composite displayed increased water absorption, reaching a saturation level of 27%, attributed to the hydrophilic nature of DW. In stark contrast, PW@Ag-O/SA demonstrated negligible water absorption, underscoring its excellent superhydrophobic properties. The self-cleaning performance of PW@Ag-O/SA was further evaluated. As shown in Figure 8e, granulated white sugar powder was evenly spread on the surface, followed by cleaning with deionized water droplets. The water droplets maintained their spherical shape and effectively removed the starch, fully restoring the clean surface of PW@Ag-O/SA. All results show that the superhydrophobic modification significantly enhances the water resistance and self-cleaning properties of balsa-derived CPCMs. These properties prevent water absorption, mitigate interfacial degradation between SA and the scaffold, and avoid encapsulation failure and SA leakage. Additionally, the self-cleaning ability ensures the removal of surface contaminants, maintaining efficient contact between the solar–thermal conversion coating and sunlight, thus preserving high solar energy conversion efficiency.

## 3. Materials and Methods

### 3.1. Preparation of Wood Morphology Genetic Starting Encapsulation Scaffold

The preparation of the wood morphology genetic starting encapsulation scaffold was performed following a method established in our previous study [52]. Balsa wood timber was first cut into thin slices measuring 10 mm × 10 mm × 3 mm, designated as RW, which were then sequentially washed with anhydrous ethanol and deionized water to remove dust and surface impurities. After oven-drying, the RW slices were immersed in deionized water overnight to ensure complete saturation, which was confirmed when all samples sank to the bottom of the container. The fully saturated wood blocks were transferred into a reaction solution containing 1 wt% NaClO_2_, with the pH adjusted to 4.6 using acetic acid, and subjected to mild boiling on a heating plate for a specified duration to completely remove the lignin. After delignification, the samples were carefully boiled and rinsed repeatedly with deionized water until the yellow-green coloration disappeared, leaving a pure white state. Finally, the delignified wood samples were frozen at −18 °C for 5 h and subsequently dried in a freeze dryer. The resulting wood morphology genetic starting encapsulation scaffold was designated as DW.

### 3.2. Catechol-Based Bionic Surface Engineering of DW

The DW was first immersed in a dopamine solution (pH = 8.5, adjusted with Tris) in a beaker, which was kept open to the atmosphere at room temperature. To ensure adequate oxygen supply, the solution was stirred for 1 min every 30 min using a glass rod, and this reaction was allowed to proceed for 24 h. After the reaction, the as-coated DW, which had transitioned from pure white to black, was removed, thoroughly washed three times with deionized water, and stored in a sealed bottle filled with water labeled as PW. The PW was then placed in a silver nitrate solution for 24 h at room temperature, and the reaction system was sealed and wrapped in aluminum foil to protect it from light. Followed by washing three times with deionized water, frozen at −18 °C for 5 h, and freeze-dried, the Ag NPs deposited balsa-derived scaffold was achieved, designated as PW@Ag. Finally, PW@Ag was added to a 10 mM ODA ethanol solution and allowed to react for 12 h at room temperature. The product was washed multiple times with ethanol and allowed to evaporate naturally, yielding the catechol-based bionic surface-engineered wood, denoted as PW@Ag-O.

### 3.3. The Formation of Balsa-Based CPCMs

Balsa-based CPCMs were prepared using a vacuum impregnation technique. Initially, solid SA was placed in a glass beaker and heated on a hot plate until it was completely melted. The aforementioned balsa-derived scaffolds were then submerged into the molten SA and transferred to a vacuum oven set at 80 °C, where a vacuum pressure of −0.1 MPa was applied. This process effectively evacuated air from the microporous and mesoporous structures, facilitating the penetration of SA throughout the entire encapsulating scaffolds. After maintaining for 1 h, the vacuum pump was switched off, and the chamber was vented with air. This vacuum–air exchange cycle was repeated four times to ensure complete impregnation of the balsa-derived scaffolds with SA. Following impregnation, the balsa-based CPCMs and excess SA mixture were placed on filter paper and dried in an 80 °C oven-dryer to remove surplus SA from the surface. The final balsa-based CPCMs were obtained when no additional SA seeped through the filter paper. The CPCMs were labeled in the format X/SA, where X represents different encapsulating scaffolds, including RW, DW, PW@Ag, and PW@Ag-O.

### 3.4. Materials and Characterization Methods

Balsa wood (*Ochroma pyramidale*) timber (10 mm × 10 mm × 500 mm) was purchased from Yidimei Model Company (Liuan, Anhui Province, China). Octadecylamine (ODA, 90%), as well as analytical-grade sodium hydroxide, stearic acid (SA), and silver nitrate, were supplied by Sinopharm Chemical Reagent Co., Ltd. (Huangpu District, Shanghai, China). Tris (hydroxymethyl)aminomethane (Tris, ACS, ≥99.8%) was procured from Macklin Biochemical Co., Ltd. (Pudong District, Shanghai, China). Dopamine hydrochloride (98.5%) was obtained from Yuanye Biotechnology Co., Ltd. (Songjiang District, Shanghai, China). Other reagents, including anhydrous ethanol, acetone, glacial acetic acid, chloroform, and benzyl alcohol, were supplied by Liyan Technology Co., Ltd. (Kunming, Yunnan Province, China). Deionized water was prepared in the laboratory.

The detailed descriptions of characterization techniques and performance testing procedures can be found in the Supplementary Materials (Note S1).

## 4. Conclusions

In this work, we successfully developed a multifunctional wood-based CPCM with superior thermal energy storage, enhanced solar-to-thermal conversion, and robust superhydrophobic properties. The innovative synthesis process involved selective delignification of balsa wood, followed by a bioinspired catechol surface modification to incorporate Ag NPs and ODA, leveraging the intrinsic anisotropy and hierarchical porosity of the wood scaffold. This design ensured effective encapsulation of SA through capillary forces and interfacial adhesion, achieving a high latent heat capacity of 175.5 J g^−1^, excellent energy storage efficiency (87.7%), and stable performance across 100 heating–cooling cycles. The integration of Ag NPs significantly improved thermal conductivity (a 2.1-fold increase compared to pure SA), enabling efficient heat transfer, while the synergistic effects of plasmonic resonance of Ag NPs and π-π stacking interactions of PDA, as well as opened unidirectional interconnected wood lumens enhanced solar-to-thermal conversion efficiency. Furthermore, the hierarchical superhydrophobic structure imparted outstanding environmental resilience, including water resistance and self-cleaning capabilities, ensuring operational stability under humid and harsh conditions. The integration of advanced functionality, environmental adaptability, and scalability makes balsa-based CPCM a promising candidate for diverse applications in solar energy harvesting, thermal management, and next-generation renewable energy technologies, opening new frontiers in sustainable energy systems.

## Data Availability

Data are contained within this article.

## Supplementary Materials

The following supporting information can be downloaded at https://www.mdpi.com/article/10.3390/molecules30010168/s1. Note S1: The Detailed Experimental Methods; Note S2: The Detailed Descriptions of Michael Addition/ Schiff base Reaction; Table S1: The phase change properties of pure SA and the CPCMs.
