# Supplementary material for: Scalable Fabrication of Light-Responsive Superhydrophobic Composite Phase Change Materials via Bionic-Engineered Wood for Solar–Thermal Energy Management
Source: Molecules. 2025 Jan 4;30(1):168. doi: 10.3390/molecules30010168 (PMC11721100; doi:10.3390/molecules30010168)
Supplement: Supplementary file 1 [file molecules-30-00168-s001.zip › molecules-3367378-supplementary.pdf]

Supplementary Materials

Scalable Fabrication of Light-Responsive Superhydrophobic Composite Phase Change Materials Via Bionic-Engineered Wood for Solar–Thermal Energy Management

Yang Meng *, Jiangyu Zhang, Yuchan Li, Hui Jiang and Delong Xie

Yunnan Provincial Key Laboratory of Energy Saving in Phosphorus Chemical Engineering and New Phosphorus Materials, Yunnan International Joint Laboratory of Sustainable Polymers, The Higher Educational Key Laboratory for Phosphorus Chemical Engineering of Yunnan Province, Faculty of Chemical Engineering, Kunming University of Science and Technology, Kunming 650500, China; zhangjiangyu@stu.edu.cn (J.Z.); liyuchan@stu.edu.cn (Y.L.); jianghui@stu.kust.edu.cn (H.J.)

* Correspondence: mengyang@kust.edu.cn

Note S1: The Detailed Experimental Methods

1.1 Characterization

The balsa-derived scaffold and the as-prepared CPCMs were sliced and mounted on conductive tape, then sputter-coated with 5 nm of Au-Pt. The samples were examined using a Field Emission Scanning Electron Microscope (FE-SEM, Sigma VP) at 10 kV to observe surface morphology. Fourier-transform infrared (FTIR) spectroscopy was performed using a Spectrum Two FTIR spectrometer in ATR mode, scanning from 500 to 4000 cm^{-1} with 32 scans and a 4 cm^{-1} resolution. X-ray diffraction (XRD) was conducted with a Rigaku smartLab diffractometer using CuKα radiation (λ = 1.541 Å), at 40 kV and 200 mA. The materials were ground into powder before testing. X-ray photoelectron spectroscopy (XPS) was performed at room temperature with an AXIS Ultra system using Al Kα radiation (1486.7 eV) to analyze surface composition. Differential scanning calorimetry (DSC, DSC 3+, METTLER TOLEDO) was used to test the phase change behavior of the materials before and after thermal cycling. A 5-10 mg sample was heated and cooled between 5 ℃ and 90 ℃ at 5 ℃/min, under nitrogen flow (50 mL/min). A preliminary heating-cooling cycle was performed to eliminate thermal errors. Thermogravimetric analysis (TGA) was conducted using a TA Q500 instrument, heating the sample under nitrogen from 30 ℃ to 700 ℃ at 10 ℃/min, with a 60 mL/min nitrogen flow. The contact angle of the modified balsawood and its composites was measured using a video contact angle goniometer (Theta Flex, Biolin Scientific). The samples, prepared into thin sheets with dimensions of 10 mm × 10 mm × 3 mm, were placed on the instrument stage. A 3 μL droplet of deionized water was dispensed onto the surface of each sample, and the contact angle was recorded at a rate of 24 frames per second.

1.2 Performance

Stability testing: for the balsa-derived scaffold, the durability of the superhydrophobic coating was tested by subjecting it to 50 tape peel cycles, ultrasonic treatment, boiling, and immersion in acetone, chloroform, and benzyl alcohol. The changes in the contact angle were recorded after each test. For the CPCMs, the stability of the superhydrophobic properties with temperature variations was tested. Samples were heated from 20 ℃ to 100 ℃ in 20 ℃ increments, and the contact angle was measured after each temperature step, as well as after 100 cycles of thermal storage. Thermal cycling stability: thermal cycling stability was tested in a xenon lamp aging chamber. Samples were placed inside the chamber, with the temperature set to fluctuate between 0° C and 100 ℃ over 100 cycles. After every 10 cycles, the samples were removed and subjected to DSC analysis. Solar thermal conversion testing: solar thermal conversion was tested using a solar simulator with an intensity of 1 sun (1000 W/m^2). As the sample was irradiated, an infrared thermography camera (T620, FLIR) was used to capture images, and a PT100 temperature sensor connected to a data logger monitored the temperature changes. Once the sample reached thermal equilibrium, the solar simulator was turned off, and the cooling process was monitored in the same manner.

Note S2: The Detailed Descriptions of Michael Addition/ Schiff base Reaction:

Michael addition: This reaction involves the nucleophilic attack of amine or thiol groups in polydopamine (PDA) on the β-carbon of α,β-unsaturated carbonyl compounds, resulting in stable covalent bonds that enhance the scaffold's functional properties.

Schiff base reaction: This process occurs through the condensation of amine groups in PDA with aldehyde or ketone groups, forming imine (C=N) linkages that contribute to the functionalization and stability of the CPCM.

Table:

Table S1. The phase change properties of pure SA and the CPCMs

**Samples**	T_m **(℃)**	ΔH_m **(KJ/kg)**	T_c **(℃)**	ΔH_c **(KJ/kg)**	E **(%)**
SA	68.40	200.1	65.63	198.8	100
RW/SA	66.54	131.7	64.82	130.8	65.8
DW/SA	67.78	168.7	65.32	167.6	84.3
PW@Ag-O/SA	67.87	175.5	65.45	174.3	87.7
